# Enhancing the Sensitivity of a Thermal Microflow Sensor: A Comprehensive Modeling and Simulation Study

**DOI:** 10.3390/mi16020231

**Published:** 2025-02-18

**Authors:** Junhua Gao, Liangliang Tian, Zhengfu Cheng

**Affiliations:** 1 School of Electronic and Information Engineering, Chongqing University of Arts and Sciences, Chongqing 402160, China; 2 School of Electronic Engineering, Heilongjiang University, Harbin 150080, China

**Keywords:** thermal microflow sensor, sensitivity, porous silicon, VO_2_(B), insulation cavity

## Abstract

The advancement of microfluidic technology has introduced new requirements for the sensitivity of microflow sensors. To address this, this paper presents a novel high-sensitivity thermal microflow sensor incorporating a heat-insulating cavity structure. The sensor utilizes porous silicon as the substrate and employs vanadium dioxide as the thermistor element. This study employed COMSOL Multiphysics finite element software 5.6 to investigate the impact of materials and structural factors on the sensor’s sensitivity, as well as considering the dynamic laws governing their influence. Additionally, the effects of thermal expansion and thermal stress on the microstructure of the sensor are thoroughly examined. The research results show that the sensitivity of the sensor was influenced by key factors such as the distance between the heater and the thermistors, the diameter of the flow channel, the power of the heater, and the presence of an insulation cavity. The utilization of B-phase vanadium dioxide, known for its high temperature coefficient of resistance and suitable resistivity, led to a significant reduction in sensor size and a remarkable improvement in sensitivity. The implementation of four thermistors forming a Wheatstone full bridge further enhanced the sensor’s sensitivity. The sensor’s sensitivity was substantially higher when employing a porous silicon substrate compared with a silicon substrate. Moreover, the integration of a micro-bridge and four micro-beams composed of silicon nitride into the sensor’s structure further improved its sensitivity. The proposed design holds promise for enhancing the sensitivity of thermal microflow sensors and offers valuable insights for future advancements in MEMS technology.

## 1. Introduction

Microfluidic systems are technologies that enable precise measurement and manipulation of fluids at the microscale [1], and they are widely applied across various industries including industrial production, medical treatment, precision electronics manufacturing, biochemical and medical fields, and process control [2,3,4]. With advancements in micro-electro-mechanical system (MEMS) processing technology, microfluidic systems based on MEMS thermal flow sensors have emerged as a significant area of research due to their broad measurement range, high sensitivity, and low limits of flow detection [5,6,7].

Traditional microflow sensors primarily utilize silicon as the substrate material to ensure compatibility with CMOS technology [8,9,10]. However, the high thermal conductivity of silicon (approximately 150 W/(m·K)) [11] leads to a notable discrepancy between the heat detected by the thermistor film and the intended heat during detection of weak signals, thereby limiting the sensor’s sensitivity. To address this issue, researchers have adopted suspended structures [12,13], although these structures compromise the robustness of the sensor and introduce additional challenges. Recent studies have proposed sensor designs that incorporate thermal insulation channels or cavities to reduce heat loss and enhance sensitivity [14,15]. In these new configurations, parallel-arranged micro-bridges are used to deposit heating resistors and thermistors; however, these sensors still face significant heat dissipation problems, resulting in most thermal flow sensors consuming over 1 mW of power.

In recent years, the integration of porous silicon technology has brought significant advancements to thermal flow sensors. The low thermal conductivity of porous silicon (approximately 0.62 W/(m·K)) [16] effectively reduces heat loss and improves the sensitivity of flow measurement, and this approach has been explored in depth in practical applications [17,18]. Furthermore, the optimization of thermistor positioning has garnered considerable attention, with studies demonstrating that the arrangement of thermistors significantly impacts the sensor’s response time and sensitivity [19,20,21].

A comprehensive overview of the evolution of microfluidic technology in the field of gas sensors has previously been provided, progressing from basic electronic techniques to more advanced optical methods such as surface-enhanced Raman spectroscopy (SERS). That article also summarized various detection approaches and highlights the advantages and disadvantages of microfluidic sensors [22]. COMSOL Multiphysics 5.6 has been used to simulate and compare simple and serpentine three-dimensional numerical models, in a study that employed multiphysics modeling of diffusion, surface adsorption/desorption, and surface reactions to investigate the impact of microchannel geometries on the performance of microfluidic gas sensors [23]. A highly sensitive and flexible piezoresistive flow sensor using vertical graphene nanosheets as the core material has been developed. This sensor exhibits high sensitivity and excellent mechanical flexibility [24]. The latest advancements in nano-coating technology for heat transfer applications are discussed, highlighting how different types of nano-coatings can enhance thermal conductivity and the importance of these coatings in improving the efficiency of thermal management systems [25]. Additionally, the transformative role of thermal metamaterials in heat transfer is explored, showcasing their potential in thermal management and energy applications [26]. A review of multiphysics modeling of thermal flow sensors highlights the impact of various physical effects on sensor performance, providing a theoretical foundation for the current research [27]. Additionally, the design of low-power microflow sensors for portable applications has been discussed, offering a comparison with the low-power objectives outlined in this study [7].

In summary, these previous studies lay a solid foundation for the ongoing development of thermal microfluidic sensors, confirming the potential of porous silicon for enhancing sensitivity while emphasizing the importance of design details in optimizing sensor performance. This paper proposes a high-sensitivity thermal sensor that combines porous silicon with vanadium dioxide and features a novel insulating cavity structure, aiming to enhance the performance of MEMS flow sensors and address the key limitations of traditional sensors, thereby providing more effective solutions for microfluidic applications across various industries.

## 2. Principles and Analysis

The proposed thermal microflow sensor operates based on Thomas’ theory, which states that the amount of heat released or absorbed by a fluid is proportional to the mass flow of the fluid [28]. When a voltage is applied to the heater in the sensor, it generates heat, causing the heater’s temperature to be higher than the ambient temperature. In the absence of fluid flow, the temperature field on the sensor’s surface exhibits a symmetrical distribution. However, when fluid flows through the sensor, it carries heat from the upstream thermistors to the downstream thermistors. This disrupts the thermal balance of the sensor and creates a temperature gradient along the direction of fluid flow. As a result, the resistance of the four thermistors changes, and the output voltage deviates from zero. By detecting the temperature difference between the two sets of symmetrically distributed thermistors, the flow rate of the fluid can be determined. To summarize, when voltage is applied to the heater, fluid flow introduces a temperature gradient that disrupts the thermal balance of the sensor. This temperature difference between the thermistors can be used to calculate the flow rate of the fluid being measured.

Heat transfer in the thermal microflow sensor can be categorized into three main types: heat conduction (*P_cond_*), heat convection (*P_conv_*), and heat radiation (*P_rad_*). However, heat radiation is typically comparatively very small and is often disregarded. As a result, the primary focus is on conduction and convection heat transfer, represented as *P ≈ P_cond_* + *P_conv_*.

Based on the first-order approximation of the heating plate boundary theory, the expressions for heat conduction and heat convection are obtained as follows [9]:

(1) $$P_{cond}=\lambda L^{2}\Delta T,$$

(2) $$P_{conv}=hL^{2}\Delta T,$$

where *L* and *L*^2^ are the size and the area of the chip, respectively, and Δ*T* is the difference between the average temperature of the sensor and the temperature of the fluid. *h* is the convection coefficient of the sensing surface, which is equal to $0.664k_{f}a^{-1/3}\mu^{-1/6}L^{-1/2}U^{1/2}$, where $k_{f}$, a, μ and U are the thermal conductivity, thermal diffusivity, kinematic viscosity, and air velocity of the fluid.

Therefore, the total power dissipation *P* of the thermal flow sensor can be written as follows:

(3) $$P=(\lambda L^{2}+0.664k_{f}a^{-1/3}\mu^{-1/6}L^{3/2}U^{1/2})\Delta T,$$

In constant power mode, the temperature difference Δ*T*_0_ between the upstream and downstream thermistors on both sides of the heater satisfies the following relationship [29]:

(4) $$\Delta T_{0}=c\frac{k_{f}L}{k_{s}D}{\left(\frac{\tau_{\omega }L^{2}}{\mu a}\right)}^{1/3}\Delta T,$$

where *c* is a quantity related to the distance between the thermal element and the heater, $k_{s}$ is the thermal conductivity of the chip, and *D* is the thickness of the chip. $\tau_{\omega }$ is the wall shear stress of the airflow surface, whose relationship with the flow velocity is determined by the surface layer, and the relationship between them depends on the geometry of the probe.

For laminar flow with a Reynolds number satisfying $R_{e}<2300$ [10], Δ*T*_0_ can be rewritten as follows [29]:

(5) $$\Delta T_{0}=K_{s}K_{p}P_{r}^{1/3}R_{e}^{1/2}\Delta T,$$

where $K_{s}$ is the geometric parameter of the sensor chip, $K_{p}$ is the parameter related to the overall thermal probe geometry, $P_{r}=\frac{\mu }{\rho a}$ is the Prandtl number of the fluid, $R_{e}=\rho \frac{Ud}{\mu }$ is the Reynolds number, ρ is the fluid density, and *d* is the diameter of the flow channel.

(6) $$\Delta T_{0}=K_{s}K_{p}{\left(\frac{\mu }{\rho a}\right)}^{1/3}{\left(\rho \frac{Ud}{\mu }\right)}^{1/2}\Delta T=K_{s}K_{p}\frac{{\left(\rho^{1/3}Ud\right)}^{1/2}}{{\left(\mu^{1/2}a\right)}^{1/3}}\Delta T,$$

**Figure 1:**
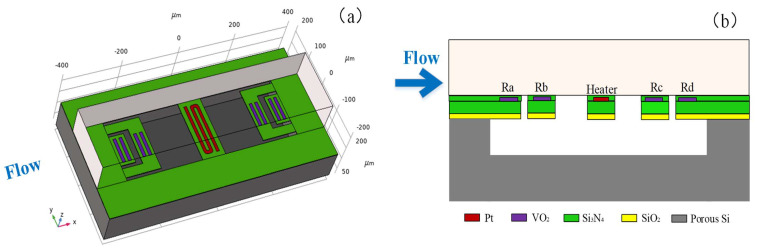
Structural diagram (**a**) and cross-sectional diagram (**b**) of the microflow sensor.

The four thermistors depicted in Figure 1a are arranged to form a Wheatstone full bridge configuration, as illustrated in Figure 1b. The output voltage of the Wheatstone bridge can be calculated as follows:

(7) $$V_{O}=\frac{R_{a}R_{b}-R_{c}R_{d}}{\left(R_{a}+R_{c})(R_{b}+R_{d}\right)}V_{s}.$$

When fluid flows through the sensor, Equation (7) becomes:

(8) $$V_{O}=\frac{\left(R_{c}+\Delta R_{c})(R_{d}+\Delta R_{d})-(R_{b}+\Delta R_{b})(R_{a}+\Delta R_{a}\right)}{\left(R_{a}+\Delta R_{a}+R_{c}+\Delta R_{c})(R_{b}+\Delta R_{b}+R_{d}+\Delta R_{d}\right)}V_{s},$$

If the flow rate is very small, in order to minimize power consumption of the sensor, the change in resistance (Δ*R*) caused by temperature becomes much smaller compared with the resistance value (*R*) before the change. Therefore, we can assume that Δ*R << R*, and Equation (8) can be rewritten as follows:

(9) $$V_{O}=\frac{R_{1}(\Delta R_{c}-\Delta R_{b})+R_{2}(\Delta R_{d}-\Delta R_{a})}{(R_{1}+R_{2}{)}^{2}}V_{s},$$

According to the definition of the temperature coefficient of resistance (*α*), we can obtain:

(10) $$\Delta R=R_{0}\alpha (T-T_{0}),$$

Substituting Equation (10) into Equation (9), we arrive at:

(11) $$V_{O}=\frac{\alpha R_{1}R_{2}(T_{c}-T_{b})+\alpha R_{1}R_{2}(T_{d}-T_{a})}{(R_{1}+R_{2}{)}^{2}}V_{s},$$

From Equation (11), it is evident that increasing the temperature difference between the upstream and downstream thermistors of the sensor enhances sensitivity. Referring to Equations (4) and (5), it can be observed that increasing the chip size, reducing the chip thickness, and minimizing lateral heat conduction contribute to an increase in the temperature difference. However, a larger chip size results in greater heat loss and higher heating power, as indicated in Equation (3). Conversely, if the chip size is too thin, its robustness is compromised and this increases susceptibility to damage. Therefore, reducing lateral heat conduction becomes the optimal choice. In constant power supply mode, employing a substrate material with lower thermal conductivity or incorporating an insulating cavity effectively reduces lateral heat conduction. This approach enables the attainment of a larger temperature field, improving the sensor’s sensitivity while simultaneously reducing power consumption.

Based on the considerations mentioned above, the structural diagram and cross-sectional diagram of the thermal microflow sensor in this paper were determined as depicted in Figure 1a,b. The sensor comprised five main components: a porous silicon substrate, a heat-insulating cavity, a microflow channel, thermistors, and a heater. The fabrication of the sensor involved its construction on a porous silicon substrate. Etching was performed on the substrate to create a heat-insulating cavity. A micro-bridge and four micro-beams made of silicon nitride were positioned above the cavity, providing support for the heater located at the center and the four thermistors. The structure of the sensor as illustrated in the diagrams enabled the reduction of lateral heat conduction, thereby enhancing its sensitivity and minimizing power consumption. The dimensions of the porous silicon substrate were 0.8 mm × 0.4 mm × 0.1 mm, while the cavity measured 0.6 mm × 0.2 mm × 0.05 mm. The dimensions of the microchannel were 0.8 mm × 0.2 mm × 0.1 mm, and the microbridge and microbeams both had a thickness of 2 µm.

## 3. Design and Simulation

**Figure 2:**
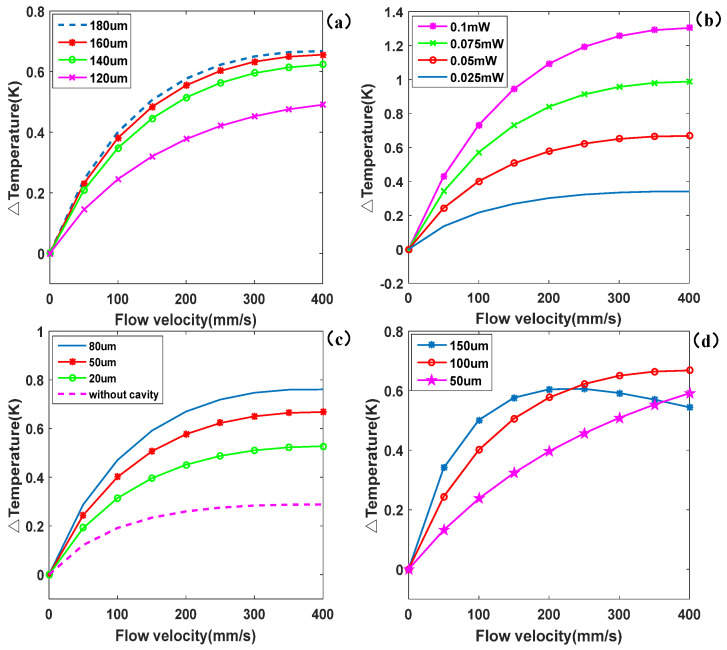
Temperature differences between the upstream and downstream thermistors changed with the flow rate: (**a**) different distances between the heater and thermistors; (**b**) heating power; (**c**) insulation cavity, and (**d**) diameter of flow channel.

The sensitivity of a sensor can also be affected by several factors, such as the distance between the heater and the thermistors, the diameter of the flow channel, and the heating power. To investigate these influences, a three-dimensional model of the thermal insulation cavity structure incorporating micro-bridges and micro-beams was created using the finite element simulation software COMSOL Multiphysics 5.6. This model enabled analysis of the sensitivity of the sensor and the variation patterns associated with the aforementioned factors. Figure 2 illustrates the structure of the thermal insulation cavity with micro-bridges and micro-beams that was used in the simulation. The model helped in understanding the impact of different variables on the sensor’s sensitivity, providing insights into the behavior and trends associated with these factors.

The positioning of the thermistors played a crucial role in maximizing the temperature difference, as depicted in Figure 2. From Figure 2a, it can be seen that at a small flow rate, increasing the distance between the thermistors and the heater enhanced the temperature difference, leading higher sensitivity of the sensor. However, as the distance continued to increase, the increase in sensitivity gradually diminished. This was because as the thermistors moved further away from the heater, their temperature fields decreased, resulting in a reduction in the temperature difference. Therefore, a larger distance does not always result in increased sensitivity. Smaller distances between the thermistors and the heater offer a larger measurement range, making them more suitable for measuring higher flow rates. On the other hand, longer distances are more prone to saturation when measuring high flow rates, despite offering greater temperature differences and higher sensitivity.

Furthermore, applying higher heating power to the heater led to increased sensitivity of the sensor, as shown in Figure 2b, because a larger heating power generated a greater temperature difference between the sensor and the ambient temperature. However, excessive power consumption can cause the fluid temperature itself to rise, which may not be conducive to the measurement and control of temperature-sensitive biological fluids in microfluidic systems.

Figure 2c shows that the microflow sensor without a cavity exhibited minimal temperature difference between the upstream and downstream thermistors at a low rate of flow. As the size of the cavity increased, the temperature difference also increased. The presence of the cavity reduced heat dissipation and lateral heat conduction, resulting in a larger difference in temperature between the sensor and the environment under the same power input. Ideally, a larger cavity size enhances the sensitivity of the sensor. However, a larger cavity also makes the chip more fragile and susceptible to damage under vibration conditions.

The results presented in Figure 2d demonstrate that within a range of low rates of flow, a larger diameter of the flow channel led to increased temperature difference and enhanced sensitivity. However, as the diameter of the flow channel increased, the measurements tended to saturate prematurely, resulting in a narrower operational range. For instance, when the flow channel diameter was 50 μm, the sensor exhibited a linear output across a flow rate range of 0–400 mm/s. In contrast, with a diameter of 150 μm, the output initially showed a linear increase but transitioned to non-linearity as the flow velocity increased, with a linear range reduced to less than 0–100 mm/s.

The increase in flow channel diameter enhanced the contact area between the fluid and the thermal resistor, thereby improving thermal exchange and resulting in a larger temperature differential. However, as the flow rate increased, this thermal exchange began to saturate, limiting any further increase in temperature difference. Conversely, narrower flow channels facilitated tighter thermal coupling between the fluid and the thermal resistor, making the temperature difference more sensitive to changes in flow rate and thereby extending the linear output range. Nevertheless, when the temperature difference reached its upper limit, the output signal saturated more rapidly.

In the context of constant power operation, it is important to note that as the flow rate increased, the temperature of the heater tended to decrease, which subsequently led to a reduced signal from the thermoelectric stack. This phenomenon was particularly pronounced in the 150 μm diameter channel. As fluid flowed through the larger channel, the increased thermal mass and diminished heating efficiency contributed to more rapid saturation of the output signal. Therefore, while larger diameters may enhance sensitivity at lower flow rates, they ultimately constrain the sensor’s performance at higher flow rates, due to thermal saturation effects. A thorough understanding of this trade-off is crucial for optimizing sensor design tailored to specific applications.

**Figure 3:**
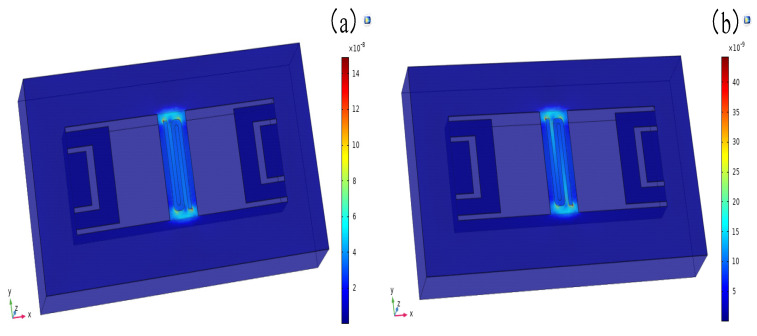
Diagram of equivalent stress distribution in the sensor micro-bridge: (**a**) *U* = 0 mm/s; (**b**) *U* = 100 mm/s.

In this thermal microflow sensor, the Si_3_N_4_ micro-bridge and micro-beams primarily serve as supports and provide heat insulation. Given the miniaturization of the device, the mechanical support capability of these components is of utmost importance in the design. Neglecting this aspect could lead to warping, deformation, and potentially cause structural damage or collapse, resulting in the device’s failure to function properly. Therefore, a comprehensive set of multi-physics simulations encompassing thermal expansion, electromagnetic heating, solid mechanics, and membrane behavior were conducted to guide the design of the microstructure. Figure 3 illustrates the distribution of equivalent stress in the micro-bridge and micro-beams of the sensor. This analysis provides insights into the stress levels and potential areas of concern within these components. By examining the stress distribution, designers can identify regions that may be prone to failure or excessive deformation. This information is crucial for optimizing the microstructure’s design to ensure its mechanical integrity and durability under operating conditions.

The Si_3_N_4_ used in the micro-bridge and micro-beams had an elastic modulus of 300 GPa, and a residual stress of 250 MPa. According to previous research [30], when the equivalent stress on the micro-bridge and micro-beams reaches 0.2% of the residual stress (0.5 MPa), the maximum stress exceeds the yield limit, resulting in permanent deformation. In Figure 3, the maximum equivalent stress in the sensor can be observed at the corner of the heater. At flow rates of 0 mm/s and 100 mm/s, the maximum equivalent stresses were measured to be 17.28 × 10^−8^ MPa and 5.17 × 10^−8^ MPa, respectively. As the flow rate increased, the temperature of the sensor decreased, resulting in a decrease in stress. Furthermore, the equivalent stress on the sensor was seven to eight orders of magnitude smaller than 0.5 MPa, indicating that it would not cause permanent deformation to the structure.

Based on these findings, it was concluded that the various components of the sensor would remain intact under the simulated heating power load. The stress levels were well below the yield limit, ensuring the ability of the micro-bridge and micro-beams to withstand the mechanical demands and maintain their structural integrity.

Overall, the parameters of the microflow sensor designed in this paper were determined as follows:

Distance: The distance between the thermistor near the heater and the heater itself was set to 180 μm.

Cavity: The depth of the heat-insulating cavity was 50 μm.

Heating power: The heating power applied to the heater was 0.05 mW.

Flow channel: The height dimension of the microflow channel was 100 μm.

The dimensions of various components are specified as follows:

Porous silicon substrate: Length, width, and thickness of 0.8 mm × 0.4 mm × 0.1 mm.

Cavity: Length, width, and thickness of 0.6 mm × 0.2 mm × 0.05 mm.

Microfluidic channel: Length, width, and thickness of 0.8 mm × 0.2 mm × 0.1 mm.

Micro-bridge and micro-beams: Both had a thickness of 2 μm.

To reduce the size of the heater, a platinum (Pt) heater with dimensions of 180 μm × 10 μm × 0.2 μm was chosen due to its low resistivity. This heater had a resistance of 30 Ω.

As shown in Figure 1a, the thermistors were deposited on the micro-beams to minimize heat conduction at the junction of the heater and the thermistors. Vanadium dioxide (VO_2_) was used as the thermistor material due to its high resistivity. To achieve a suitable resistance value, the two resistance bars illustrated in Figure 1a were connected in parallel to form the upstream and downstream thermistors. When no voltage was applied to the heater (at room temperature), the resistance values of the upstream and downstream thermistors were approximately 1500 Ω.

The four thermistors were divided into two groups, i.e., *R*_a_ and *R*_d_, *R*_b_ and *R*_c_, as shown in Figure 1b. These four thermistors constituted the four arms of the Wheatstone bridge. The distances between the two sets of thermistors and the heat source were 140 μm and 210 μm, respectively. The thermistors were protected by a thin layer of insulating silicon nitride.

These specific parameters and configurations were selected based on the simulation results to optimize the sensitivity and performance of the microflow sensor designed in this study.

## 4. Results and Discussion

**Figure 4:**
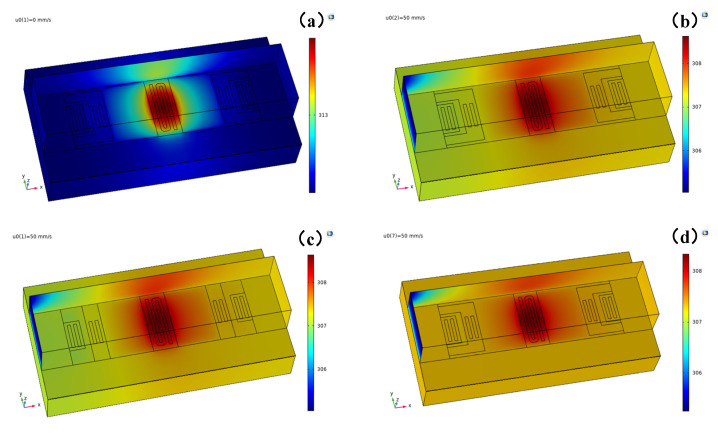
The temperature field distribution of sensor #1 at *U* = 0 (**a**) and *U* = 50 mm/s (**b**); sensor #2 (**c**) and sensor #3 (**d**) at *U* = 50 mm/s.

Fluid–solid thermal coupling simulation of the designed sensor was conducted to assess its performance. The heater was subjected to a constant power of 0.05 mW, and the gas used was air. The inlet air temperature was set to 293.15 K, and the outlet pressure was set to 0. To enable comparison, we designed three different sensor prototypes, as shown in Figure 4. The key differences between the three sensors are described in Table 1. The temperature for each section of sensor #1, sensor #2, and sensor #3, both with no fluid flow and at a flow velocity of 50 mm/s, are provided in Table 2.

It was observed that at a heating power of 0.05 mW, the highest temperature recorded by the sensor was 313.67 K, while the average temperature was 312.48 K. Considering that the ambient temperature was 293.15 K, this was a temperature difference of 19.33 K. Furthermore, in sensor #1, when the flow rate was 0, the temperature field surrounding the heater exhibited a symmetrical distribution and the temperatures of the upstream and downstream thermistors were equal. However, at a flow rate of *U* = 50 mm/s, the average temperature of the sensor decreased to 307.35 K, indicating that a portion of the heat was carried away by the flowing fluid. Notably, the temperature of the downstream thermistors was higher than that of the upstream thermistors. For instance, the temperature difference between the upstream and downstream thermistors in the first group was 0.3 K, while that in the second group was 0.25 K. This disparity confirmed that thermistors located further from the heater exhibited a higher temperature difference.

Furthermore, when *U* = 50 mm/s, the average temperatures of the three sensors were the same. However, sensor #3, which featured a silicon substrate, exhibited a lower maximum temperature compared with sensor #1 and sensor #2 with porous silicon substrates. Additionally, the average temperature differences between the upstream and downstream thermistors in sensor #3 were only 0.02 K and 0.05 K, significantly lower than the values observed in sensor #1 and sensor #2. This discrepancy arose due to the higher thermal conductivity of silicon (150 W/(m∙K)) compared to porous silicon (0.62 W/(m∙K)), resulting in reduced lateral heat conduction and smaller differences in temperature between the upstream and downstream thermistors in sensor #3.

Moreover, sensor #1 and sensor #2 both employed porous silicon substrates. However, sensor #1, which utilized a single-bridge and four-micro-beams structure, exhibited greater differences in temperature than sensor #2. For example, at a flow rate of *U* = 50 mm/s, the temperature differences between the upstream and downstream thermistors of sensor #2 were 0.29 K and 0.2 K, respectively, lower than those of sensor #1. This discrepancy can be attributed to the micro-bridges in sensor #2 bringing the heater and thermistors closer to each other, resulting in increased lateral heat conduction.

**Figure 5:**
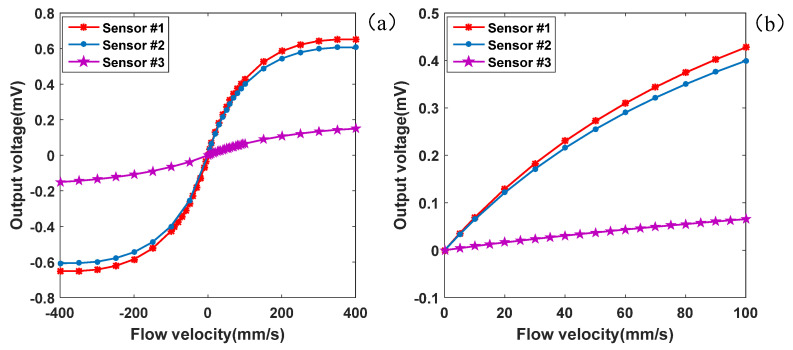
Change in output voltage of the microflow sensor with the flow rate (**a**) and change in the area of low flow rate (<100 mm/s) (**b**).

COMSOL Multiphysics software 5.6 was utilized to simulate the thermal fluid–solid electric multiphysics coupling of the three sensors in the flow velocity range of 0–400 mm/s. The simulation incorporated various physical field components, including solid and fluid heat transfer, laminar flow, current in the shell, and electrical circuits. The multiphysics fields encompassed non-isothermal flow and electromagnetic heating. The current component in the shell applies a power of 0.05 mW to the heater. VO_2_ was employed, chosen for its higher temperature coefficient of resistance (TCR) and resistivity compared with metal materials. To meet the operational requirements, a power supply voltage of 0.05 V was provided to the Wheatstone bridge using circuit components. Figure 5 displays the change curve of the output voltage of the three sensors with the flow rate. It is important to note that the output voltage is not amplified in the figure. Through these simulations, a comprehensive understanding of the sensors’ performance and variation in output voltage with different flow rates could be obtained. The multiphysics coupling analysis allowed a holistic assessment of the sensors’ functionality, considering the influence of fluid dynamics, heat transfer, and electrical circuitry on the output voltage. The results for sensor #1, sensor #2, and sensor #3 are presented in Table 3.

The output voltage of the three sensors increased with the flow rate in the range of 0–400 mm/s, as depicted in Figure 5. With a low rate of flow (<100 mm/s), the voltage exhibited a linear relationship with the flow rate. However, as the flow rate continued to rise, the voltage increase gradually diminished and approached saturation. Consequently, the linear output range of the sensors was 0–100 mm/s.

Moreover, at the same flow rate, sensor #1 exhibited slightly higher output voltage compared with sensor #2 and a significantly higher output voltage than sensor #3. This indicates that the sensor with a porous silicon substrate possessed higher sensitivity compared to the one with a silicon substrate. This sensitivity difference became more prominent as the flow rate increased. For instance, at a flow rate of 50 mm/s, the output voltages of sensor #1, sensor #2, and sensor #3 were 0.272 mV, 0.255 mV, and 0.037 mV, respectively. Similarly, at a flow rate of 100 mm/s, these values were 0.427 mV, 0.399 mV, and 0.066 mV, respectively. The linear output range of the microflow sensors spanned from 0 to 100 mm/s. Within this range, the sensitivities of sensor #1, sensor #2, and sensor #3 were 4.27 mV/(m/s), 3.99 mV/(m/s), and 0.66 mV/(m/s), respectively. Considering that the sensor heater operated at a constant power of 0.05 mW, the standardized sensitivities of the three sensors were 85.40 mV/(m/s)/(mW), 79.8 mV/(m/s)/(mW), and 13.2 mV/(m/s)/(mW). These results demonstrate that using a porous silicon substrate with low thermal conductivity effectively enhanced the sensor’s sensitivity. Additionally, placing the thermistors on the micro-beams, which were further away from the heater than those on the micro-bridges, reduced the heat conduction between the heater and the thermistors, further improving the sensor’s sensitivity.

Furthermore, the cross-sectional area of each sensors’ flow channels was 0.1 mm × 0.2 mm = 0.02 mm^2^. As a result, the measurement range of the flow sensors spanned from 0 to 120 μL/min. For instance, at a flow rate of 6 mm/s, the output voltage was 42.7 μV, and at 6.2 mm/s, the output voltage was 44.1 μV, resulting in an increase of 1.4 μV. Similarly, at 6.4 mm/s, the output voltage was 45.4 μV, with an increase of 1.41 μV. Thus, the flow rate detection resolution of this sensor was below 0.24 μL/min.

In this study, the sensitivity of the thermal flow sensor based on a porous silicon substrate was measured at 4.27 mV/(m/s), significantly higher than the value previously reported [31], which was 0.603 mV/(m/s). The normalized sensitivity of our sensor was 85.40 mV/(m/s)/(mW), compared with 62.9 mV/(m/s)/(mW) [31], 60.6 mV/(m/s)/(mW) [31], 30.7 mV/(m/s)/(mW) [32], and 30.2 mV/(m/s)/(mW) [33], demonstrating a notably higher normalized sensitivity.

Moreover, through the optimization of materials and structural design, our sensor effectively reduces power consumption by incorporating a thermal insulation cavity structure and high-performance materials such as vanadium dioxide. The power consumption of our sensor was 0.05 mW, significantly lower than values of 10 mW [32] and 25 mW [34] for similar sensitivity levels. This reduction in power consumption not only enhances the operational efficiency of the sensor but also makes it more suitable for portable applications.

The flow rate range of the sensor in this study extended from 0 to 120 μL/min, with a flow detection resolution below 0.24 μL/min, demonstrating high precision in microfluidic measurements. In comparison to the existing literature, which reports flow ranges of 0 to 1500 μL/min [31] and 0 to 1800 μL/min [32], it is important to note that while the range of our flow rate was narrower, this was a result of our specific design requirements. Nevertheless, these results indicate that our sensor design holds greater potential for flow measurement applications.

In summary, this study aimed to significantly enhance the sensitivity and reduce the power consumption of MEMS flow sensors through the use of innovative materials and structural design. These advancements not only address the limitations of traditional sensors documented in the literature but also provide a more efficient technological foundation for future microfluidic applications, underscoring their important research and application value.

## 5. Conclusions

The thermal fluid–solid electric multiphysics coupling characteristics of three microflow sensors were investigated using COMSOL Multiphysics 5.6 simulations within a flow rate range of 0–400 mm/s. The results showed that at a low rate of flow (<100 mm/s), the output voltage of the sensors exhibited a linear relationship with the flow rate. As the flow rate increased, the increase in voltage gradually slowed down and approached saturation, indicating a linear output range of 0–100 mm/s. At the same flow rate, the sensor with a porous silicon substrate (sensor #1) demonstrated a slightly higher output voltage compared with the sensor with a silicon substrate (sensor #2), and significantly higher output voltage than the sensor without the heat-insulating cavity structure (sensor #3), indicating that the use of a porous silicon substrate with low thermal conductivity effectively enhanced the sensor’s sensitivity. Moreover, placing the thermistors on the micro-beams, which were further away from the heater than those on the micro-bridges, reduced the heat conduction between the heater and the thermistors, further improving the sensor’s sensitivity. Within the linear output range of 0–100 mm/s, the sensitivities of sensor #1, sensor #2, and sensor #3 were 4.27 mV/(m/s), 3.99 mV/(m/s), and 0.66 mV/(m/s), respectively. Considering a constant heater power of 0.05 mW, the standardized sensitivities of the three sensors were 85.40 mV/(m/s)/(mW), 79.8 mV/(m/s)/(mW), and 13.2 mV/(m/s)/(mW). The cross-sectional areas of the sensors’ flow channels were 0.1 mm × 0.2 mm = 0.02 mm^2^, resulting in a measurement range of 0 to 120 μL/min and a flow rate detection resolution below 0.24 μL/min. These findings demonstrate the significance of utilizing a porous silicon substrate and optimizing the thermal conduction path to enhance the performance of thermal microflow sensors.

## Figures and Tables

**Table 1:** The key differences between the three sensors.

Sensor	Substrate Type	Structural Details
Sensor #1	Porous Silicon	One heater, two thermistors on four micro-beams
Sensor #2	Silicon	One heater, two thermistors on three micro-bridges and two micro-beams
Sensor #3	Silicon	One heater, two thermistors on four micro-beams

**Table 2:** Temperature of sensor #1, sensor #2, and sensor #3.

T(K)		Max	Average	Up1	Down1	Up2	Down2
#1	*U* = 0 mm/s	313.67	312.48	312.37	312.37	312.46	312.46
	*U* = 50 mm/s	308.62	307.35	307.09	307.39	307.22	307.47
#2	*U* = 50 mm/s	308.62	307.35	307.10	307.39	307.26	307.46
#3	*U* = 50 mm/s	308.33	307.35	307.32	307.34	307.35	307.40

**Table 3:** Results for sensor #1, sensor #2, and sensor #3.

Parameter	Sensor #1	Sensor #2	Sensor #3
Output Voltage (50 mm/s)	0.272 mV	0.255 mV	0.037 mV
Output Voltage (100 mm/s)	0.427 mV	0.399 mV	0.066 mV
Sensitivity (mV/(m/s))	4.27	3.99	0.66
Standardized Sensitivity (mV/(m/s)/(mW))	85.40	79.8	13.2
Linear Output Range (mm/s)	0–100	0–100	0–100
Flow Measurement Range (μL/min)	0–120	0–120	0–120
Flow Detection Resolution (μL/min)	<0.24	<0.24	<0.24
Heating Power (mW)	0.05	0.05	0.05
Flow Channel Cross-Sectional Area (mm^2^)	0.02	0.02	0.02

## Data Availability

The original contributions presented in the study are included in the article, further inquiries can be directed to the corresponding author.
